# Impact of picture archiving communication systems on rates of duplicate imaging: a before-after study

**DOI:** 10.1186/1472-6963-8-234

**Published:** 2008-11-12

**Authors:** John J You, Lingsong Yun, Jack V Tu

**Affiliations:** 1 Department of Medicine, McMaster University, 1200 Main Street West, HSC-3V51, Hamilton, Ontario, L8N 3Z5, Canada; 2 Institute for Clinical Evaluative Sciences, G106-2075 Bayview Avenue, Toronto, Ontario, M4N 3M5, Canada

## Abstract

**Background:**

Electronic health information systems, such as picture archiving communication systems (PACS), are commonly believed to reduce the need for duplicate testing. However, empirical data to support this belief are not available.

**Methods:**

Before-after study using administrative claims data from the Ontario Health Insurance Plan to determine whether the introduction of PACS at 10 hospitals in the Thames Valley region of southwestern Ontario, Canada between June 2004 and December 2005 reduced the frequency of duplicate imaging examinations. The imaging modalities studied were: chest and abdominal X-ray; computed tomography of the abdomen/pelvis, head, and chest. The frequency of duplicate testing was examined at 3 different time frames: 7 days, 30 days, and 60 days after a given index test.

**Results:**

Overall frequencies of duplicate imaging were: 2.7% within 7 days of an index imaging test, 6.7% within 30 days, and 9.8% within 60 days. Comparing the 12 months before and 12 months after PACS, absolute reductions in the frequency of duplicate X-rays using 7-day, 30-day, and 60-day time frames were: 0.2% (P = 0.01), 0.6% (P < 0.001), and 0.9% (P < 0.001), respectively. In contrast, there were absolute *increases *in the frequency of duplicate CT scans after PACS of 0.0% (P = 0.92), 0.5% (P = 0.01), and 0.5% (P = 0.01), respectively.

**Conclusion:**

The frequency of duplicate imaging is relatively low and we did not find large reductions in duplicate imaging after the introduction of PACS. Independent evaluation of electronic medical systems should be conducted to confirm widely held beliefs of their potential benefits.

## Background

Picture archiving and communication systems (PACS) are computer networks dedicated to the storage, retrieval, and presentation of images produced by medical imaging devices. PACS replaces film archives and provides new capabilities such as the ability to access imaging information from various physical locations simultaneously and off-site viewing by clinicians and radiologists. PACS is commonly believed to reduce the number of unnecessary duplicate imaging tests ordered because original images are lost, misfiled, or stored at a remote location. If true, this would not only have important implications for decision-makers contemplating PACS implementation, but also for population health, given recent concerns about the potential risks of cancer from exposure to radiation during computed tomography scanning.[1]

Very few studies have evaluated the reduction in redundant imaging associated with PACS implementation. A survey of clinicians conducted at Hammersmith Hospital, United Kingdom revealed that 78% of respondents had a problem with unavailability of inpatient images before PACS versus only 14% after PACS. Forty percent of respondents indicated that they ordered one or more repeat examinations per month because of unavailable images before PACS versus only 12% after PACS.[2,3] More recently, in a survey of British Thoracic Society members, 71% of respondents agreed that there were fewer lost images when using PACS. However, the survey did not ask directly about the impact of PACS on the ordering of redundant imaging tests.[4] Studies evaluating other aspects of PACS have again almost exclusively used survey- or interview-based methods to determine users' perceptions about PACS and have generally found very high levels of user satisfaction with many different aspects of PACS.[2,3,5-11] While these studies provide valuable insight into user perceptions, they rely on users' subjective and potentially biased interpretations of their experience with PACS. To our knowledge, few empirical data are available to support these perceptions. Accordingly, we conducted a before-after study using administrative health insurance claims data to determine whether the introduction of PACS was associated with a reduction in the frequency of duplicate imaging examinations.

## Methods

### Setting

The Thames Valley Digital Imaging Network project was an $18 million (Canadian dollars) [CAD]) initiative that implemented PACS at 10 hospitals in southwestern Ontario between June 2004 and December 2005. For this study, we considered PACS to have been fully implemented when a hospital officially began "filmless" operations. These dates, along with the annual imaging volumes of each hospital, are listed in Table 1.

**Table 1:** Thames Valley Imaging Network

**Hospital**	**Annual number of X-ray examinations**	Annual number of CT examinations*	**PACS introduced (filmless date)**
**A†**	-	-	Jun 1, 2004
**B‡**			Feb 1, 2005
**C‡**	43609	36016	Mar 1, 2005
**D‡**			Mar 1, 2005
**E**	5071	-	Jun 1, 2005
**F**	1297	-	Aug 1, 2005
**G**	4007	-	Aug 1, 2005
**H**	2919	-	Sep 1, 2005
**I**	8501	5773	Oct 1, 2005
**J**	6924	3599	Dec 1, 2005

Abbreviations: PACS, picture archiving communication systems. X-ray counts are for chest and abdomen; computed tomography counts are for abdomen/pelvis, head, and chest, and were obtained using Ontario Health Insurance Plan (OHIP) data from the 2005/2006 fiscal year (April 1, 2005 to March 31, 2006).

*Computed tomography services were not provided by all Thames Valley hospitals during the observation period of this study.

†Diagnostic imaging exams were not billed to OHIP during the observation period of this study. Therefore, this institution was excluded.

‡These three hospital sites are part of the same corporation and are identified with a single institution number in the OHIP database. For the purposes of this study, hospitals B-D were treated as a single institution with a filmless date of March 1, 2005.

### Study design

We conducted a before-after study to examine changes in the frequency of repeat diagnostic imaging associated with the introduction of PACS in Thames Valley hospitals. We studied the following imaging modalities: chest X-ray, abdominal X-ray, computed tomography (CT) of the abdomen/pelvis, CT of the head, and CT of the chest. Spine X-ray, abdominal ultrasound, and magnetic resonance imaging were also considered for inclusion in this study, but were excluded since preliminary analyses revealed extremely low rates of repeat testing for these modalities (data not shown). This study received full approval from the Sunnybrook Health Sciences Centre Research Ethics Board.

### Data sources

Diagnostic imaging services in Ontario are provided without patient user fees. Ontario radiologists receive fee-for-service payment from the Ministry's Ontario Health Insurance Plan (OHIP) for outpatient X-ray examinations, and for both inpatient and outpatient CT examinations. Imaging examinations performed at Hospital A (Table 1) were not billed to OHIP during the observation period of this study; therefore, this hospital was excluded. From the OHIP database, we identified claims paid for imaging examinations using the following fee codes: chest X-ray, X090, X091, X092; abdominal X-ray, X100, X101; CT abdomen/pelvis, X126, X409, X410, X231, X232, X233; CT head, X188, X400, X401, X402, X405, X408; and, CT chest, X125, X406, X407.[12] We used the institution number associated with each claim to identify imaging exams performed at Thames Valley institutions.

### Outcome measure

We used the change in the frequency of duplicate imaging examinations after the introduction of PACS as our primary outcome measure. For the purposes of this study, a duplicate examination was defined as the occurrence of the same imaging examination (i.e., identical OHIP fee code) within a specified time frame after the index test. Since the OHIP database does not contain information about the clinical indications for diagnostic imaging tests, duplicate examinations–as defined in this study–include both unnecessary *and *clinically warranted duplicate examinations. We assumed that the frequency of clinically warranted duplicate examinations was stable before and after the introduction of PACS and that, as a result, any observed change in the frequency of duplicate testing after PACS was attributable to a change in the frequency of *unnecessary *duplicate examinations (e.g., those ordered because original images were lost, misfiled, or at a remote location).

### Statistical analysis

For each institution and each imaging modality of interest, we identified the number of index imaging tests that occurred at that institution during the 12 months before and 12 months after the introduction of PACS. Next, we determined the number of index tests associated with a duplicate test at that institution within a specified time after the index test (7 days, 30 days, or 60 days). Duplicate tests were not eligible to be counted as index tests. The frequency of duplicate testing was expressed as the proportion of index tests associated with a duplicate test. For each imaging modality, we compared the frequency of duplicate testing in the 12 months before PACS (combined across all institutions) to the frequency of duplicate testing in the 12 months after PACS (combined across all institutions) using a chi-squared test. P values less than 0.05 were considered statistically significant.

## Results

The frequencies of duplicate imaging are presented in Table 2. Overall, duplicate imaging, for X-ray and CT combined, was a relatively infrequent phenomenon with frequencies of 2.7% within 7 days of index imaging, 6.7% within 30 days, and 9.8% within 60 days.

**Table 2:** Frequency of within-institution duplicate imaging 12 months before and 12 months after introduction of PACS

	**Frequency of duplicate imaging**		
	**Before PACS**	**After PACS**	**P**
**Duplicate test within less than 7 days**			
			
*X-ray*			
Chest	1146/60598 (1.9)	1070/60366 (1.8)	0.12
Abdomen	339/8512(4.0)	273/8355 (3.3)	0.01
Total	1485/69110 (2.2)	1343/68721 (2.0)	0.01
			
*Computed tomography*			
Abdomen/Pelvis	275/14213 (1.9)	351/16060 (2.2)	0.13
Head	1099/16523 (6.7)	1180/18102 (6.5)	0.62
Chest	78/6627 (1.2)	120/8173 (1.5)	0.13
Total	1452/37363 (3.9)	1651/42335 (3.9)	0.92
			
**Duplicate test within less than 30 days**			
			
*X-ray*			
Chest	3948/58212 (6.8)	3592/58204 (6.2)	<0.001
Abdomen	667/8248 (8.1)	595/8123 (7.3)	0.07
Total	4615/66460 (6.9)	4187/66327 (6.3)	<0.001
			
*Computed tomography*			
Abdomen/Pelvis	596/13969 (4.3)	786/15764 (5.0)	0.003
Head	1640/16364 (10.0)	1856/17868 (10.4)	0.26
Chest	200/6525 (3.1)	309/8019 (3.9)	0.01
Total	2436/36858 (6.6)	2951/41651 (7.1)	0.01
			
**Duplicate test within less than 60 days**			
			
*X-ray*			
Chest	6032/56841 (10.6)	5519/56902 (9.7)	<0.001
Abdomen	857/8143 (10.5)	789/8012 (9.9)	0.16
Total	6889/64984 (10.6)	6308/64914 (9.7)	<0.001
			
*Computed tomography*			
Abdomen/Pelvis	968/13679 (7.1)	1174/15461 (7.6)	0.09
Head	1883/16257 (11.6)	2157/17736 (12.2)	0.10
Chest	341/6405 (5.3)	498/7857 (6.3)	0.01
Total	3192/36341 (8.8)	3829/41054 (9.3)	0.01

Abbreviations: PACS, picture archiving communication systems.

All numbers reported as no. of index tests associated with a duplicate test/total no. of index tests (%).

All P values are for difference in frequency of duplicate imaging before versus after PACS.

For X-ray examinations, we observed statistically significant reductions in the frequency of duplicate testing after PACS using 7 day, 30 day, and 60 day time frames to identify duplicate tests. Although relative reductions in duplicate testing were 9.1%, 8.7%, and 8.5%, respectively, these translated into numerically small absolute reductions in duplicate testing of 0.2%, 0.6%, and 0.9%, respectively.

For CT, we did not observe any reduction in duplicate testing after PACS using a 7 day time frame and in fact observed statistically significant *increases *in the frequency of duplicate CT examinations after PACS using 30 day and 60 day time frames. Once again, although relative increases were 7.6% and 5.7%, respectively, the absolute increases were numerically small (absolute increase of 0.5% in each case).

## Discussion

There is a common belief that the introduction of electronic health information systems, such as PACS, will reduce the need for duplicate testing.[2,3] For instance, the majority (58%) of PACS users, including clinicians and radiologists in Thames Valley, believe that the introduction of PACS at their institution was associated with a reduction in the number of unnecessary duplicate imaging tests (unpublished Canada Health Infoway survey data). However, empirical data supporting these beliefs have not been previously available. To our knowledge, this study is the first to report objective data regarding the impact of PACS on the frequency of duplicate testing.

Overall, we found that duplicate testing was a relatively infrequent phenomenon, with a maximum frequency of about 10% within 60 days of an index imaging examination. Since many of these duplicates would be expected to have been clinically indicated and since PACS is only hypothesized to reduce unnecessary duplicate examinations (e.g., due to unavailability of original images), it is not surprising that we only observed small absolute reductions in the frequency of duplicate X-ray examinations after the introduction of PACS. To put our findings into context, using the largest reduction observed in our study (0.9% absolute reduction in the frequency of duplicate X-ray examinations after PACS) and based on annual X-ray volumes in the Thames Valley institutions (approximately 70,000 OHIP claims for chest X-rays and abdominal X-rays in the 2005/2006 fiscal year) and an average fee per X-ray examination of approximately $30 (CAD),[12] PACS might be expected to result in 630 fewer duplicate chest or abdominal X-ray examinations in the year after the introduction of PACS or a cost-savings to OHIP of $18,900 (CAD).

We observed an unexpected increase in the frequency of CT examinations after PACS using 30 day and 60 day time frames to identify duplicates. It is possible that improved image availability after PACS may have encouraged physicians to more frequently order repeat imaging tests to monitor response to treatment or to follow-up previously detected imaging abnormalities. It is also possible that, independent of PACS, the frequency of duplicate CT imaging was increasing during the observation period of this study, thus masking a true effect of PACS in reducing the frequency of redundant examinations. Indeed, previous analyses of OHIP data suggest that this may have occurred, with much more marked increases in the prevalence of repeat CT imaging over the past decade in Ontario (58% increase between 1996 and 2004) compared to plain chest x-ray examinations (6% increase between 1996 and 2004).[13]

Reducing duplicate testing, however, is only one of many potential benefits of PACS. Previous survey- and interview-based evaluations of PACS have examined users' perceptions about PACS and have generally found high levels of user satisfaction and have reported perceptions of more efficient time utilization[6,8,10], reduced turn-around time from image acquisition to reporting[6,8], and good value for money[5]. These potential benefits are important from a health services perspective and warrant further evaluation. Indeed, the mismatch between perceived and measured benefits found in our study underscores the need for rigorous, independent evaluation of objective outcome measures to determine the benefits of new health information technologies.

This study has some limitations. First, a strength of our before-after study design was that each institution served as its own control. However, this design also assumes that the frequency of clinically warranted duplicate imaging was stable before and after PACS and that any observed change in the frequency of duplicate imaging was due to a reduction in unnecessary duplicates after PACS. It is possible that contextual increases in the frequency of duplicate testing over time may have masked true reductions in duplicate imaging due to PACS, as may have been the case for increases in duplicate CT imaging that we observed after PACS. Second, the OHIP database does not contain the reason why a given imaging test was ordered. Therefore, we could not discriminate between imaging tests repeated for clinical reasons versus those repeated because of image unavailability. More resource intensive evaluations using chart review or a prospective survey of physicians at the time of ordering could be used to address this limitation. However, since we found that baseline rates of duplicate testing are relatively low, it is unlikely that large absolute reductions in redundant imaging would be found using any methodology.

## Conclusion

In conclusion, we found that the overall frequency of duplicate imaging examinations is relatively low and we did not find large absolute reductions in the frequency of duplicate imaging examinations after the introduction of PACS. Independent evaluation of electronic medical systems should be conducted to confirm widely held beliefs of their potential benefits.

## Competing interests

The authors declare that they have no competing interests.

## Authors' contributions

JJY designed the study, interpreted the data and drafted the manuscript. LY performed the analyses. JVT participated in the design of the study, helped to interpret the data and revised the manuscript. All authors read and approved the final manuscript.

## Pre-publication history

The pre-publication history for this paper can be accessed here:


